# Quantitative interactions between *Candida albicans* and the *mutans streptococci* in patients with Down Syndrome

**DOI:** 10.4317/medoral.23162

**Published:** 2020-11-28

**Authors:** Alfredo G. Linossier, Benjamín Martinez, Carlos Y. Valenzuela

**Affiliations:** 1 Department of Dentistry and Oral Maxilo facial Surgery, School of Medicine, Pontificia Universidad Católica de Chile; 2 Oral Pathology, School of Dentistry, Faculty of Sciences, Universidad Mayor. Santiago, Chile; 3 Faculty of Medicine, Institute of Biomedical Sciences. Human Genetics Program. Universidad de Chile

## Abstract

**Background:**

Oral microorganisms produce damage through the transfer to bloodstream, colonizing other tissues or direct damage in the oral cavity. Aim to study the quantitative interactions between *C. albicans* and the *mutans streptococci* and ms serotypes in the saliva of the oral cavity of patients with Down syndrome (DS).

**Material and Methods:**

Included 120 patients of both genders, 60 patients with Down syndrome (DS) and 60 patients as a control group (CG). Samples of saliva were taken, and bacteria and fungi were grown on TYCSB and Saboureaud agar. Microbiological, serological and quantitative analyses were performed to determine the kind of isolated of microorganisms corresponding to the ms c, e, f and k for species *S. mutans* and d and g for *S. sobrinus* and *C. albicans*. Electronic scanning microscopy was employed to visualize and confirm the colonies under study. Statistics analysis included t-test proofs for matched data test, Scheffé and ANOVA.

**Results:**

Forming units (CFU) per mL of saliva of *C. albicans* a significant difference was observed among DS>CG groups. A correlation of the *C. albicans* quantity and the ms count was found by age intervals however, tendencies were different in SD and CG. Also, the CFU of *C. albicans* was different among the serotypes of ms (c, e, f, k <d, g, h, <notyped).

**Conclusions:**

These results show a significant non-random association between these two commensal microorganisms in different patient groups.

** Key words:**Down Syndrome, Candida albicans, microorganism interactions, mutans streptococci and oral cavity.

## Introduction

The oral cavity is colonized by different microorganisms in their different structures such as mucosa, teeth and saliva (1). These microorganisms constitute the biofilm, in which they grow and develop depending on the conditions of the environment if it is favorable to him and of the conditions given by the same microorganisms and the guest (2). Among these are the metabolic factors of the host and the microorganisms, chemical signals (quorum sensing) typical of them and the toxins they produce. The oral cavity research is oriented towards a model of interaction between the microorganisms that make up the community (3). During the first stages of the formation of the biofilm, bacterial cells are organized in plantonic phase rushing the surfaces of the oral cavity or indirectly bind to other bacterial cells that have already been colonized (4). This last point is confirmed by a population study of ancestral dental calculus, which shows different morphotypes of microorganisms (5).

 In research of congregation between yeasts and oral bacteria, there has been evidence of the presence *in vitro* of streptococci and *C. albicans* (6). In other studies that have used a specific fluorescein-labeled oligonucleotides probe (FITC), they allowed investigators to observe a congregation through microscopy, visualizing as a maize corn cob form where the center was occupied by *C. albicans* surrounded by species of streptococcus (7).

In a study model *in vivo*, the possible association was shown between *S.mutans* and *C.albicans* in caries production in children at an early age (8). The *C.albicans* is part of the biofilm, giving to it the modulation of the different species in terms of the quality and quantity of them that make up micro environment of the oral cavity (9).

Thus we will study the interaction between the serotypes of *mutans streptococci* (ms) and fungus *Candida albicans*, as secondary colonizers of the biofilm. They have been described as alterations in the oral cavity, and from there they can produce disturbances at the systemic level by these microorganisms. So we have the ms that includes different species in the human and within it the *S. mutans*, where it is possible to recognize serotypes such as c, e, f and k for *S. mutans* and d, g, h, for *S.sobrinus* (10). We know that ms has a great prevalence in the oral human cavity, with a range of detection for *S. mutans* between 74 and 90% while for *S.sobrinus* the prevalence is lower (11). C albicans and *S.mutans* are considered commensal microorganisms, coexisting as homeostatic mutualism. When the balance is altered, due to environmental changes occurring in the mouth or by guest alterations at the systemic level, they become pathogens (12). So, we have that the *S. mutans* is a microorganism associated with tooth decay (11). In relation to dental caries in patients with Down Syndrome the evidence is limited and controversial (13). At a systemic level the oral streptococci produces infectious endocarditis 20% (14). It affects 71% the endocarditis subacute presenting serotype k, retrieved from the heart valves (15). This possesses proteins (CBPs) approximately 120 KDa, named cnm and cbm. These surface antigens relate to the adhesion of the *S.mutans* to collagen tissue (16). As to *C. albicans*, its virulence is determined by its adherence to the surfaces of internal tissues in 5% , which occurs in several stages; the infection is due to the presence of a protein ALS3 (17). It should be kept in mind that patients with Down syndrome show multiple systemic deficiencies. In the oral cavity, both humoral and cellular immunologic responses are involved, the latter being the most impaired of the cell-mediated immune system, leading to increased autoimmune diseases (18,19). Previous studies have shown a quantitative relationship of the commensalism between *S. mutans* and *C. albicans* and the serotypes distributed between patients with DS and CG (20,21).

The objective is to quantify the interaction in the saliva of *C. albicans* and the *mutans streptococci* in patients with DS and CG.

## Material and Methods

- Subjects

During 2000 and 2001.This study was carried out in 120 males and females children and adolescent, aged 5 to19 years, were studied. The belonged to the public schools of the southern of the Metropolitan Region of Santiago.Among them 60 presented Down Syndrome (DS) and 60 were control group (CG). These data were obtained by observing ethical protocols at the time of collection. Patients who received antibiotics for less than 21 days before examination were excluded.

- Saliva samples

Were collected according to the following protocol: Two hours after breakfast a dentist brushed the patient's teeth for thirty seconds. Salivary flow was stimulated by applying a 1% solution of citric acid on the dorsal side of the tongue. After one minute, each patient's samples were collected using a glass funnel and kept at 0 º C for microbiological analysis. The volume of saliva collected was at least 0.5 ml (20).

- Microbiological samples

The saliva is homogenized in a Vortex mixer (Max MixII tipo 37600 Mixer) for 60 s; 100 μl of saliva were added to 900 μl of a Na2 HPO4 0,2M buffer solution (Sigma, St Luis,MI, EEUU)). The resulting solution was again homogenized by sonication for 2 min at 37ºC and a volume of 100 μl were streaked in an agar plate with TYCSB (22). The plates were incubated using an anaerobic system (Gas Pack jars) with a mixture of 95% N2, 5 % CO2, for 48 h at 37ºC.

The colonies were counted according to the method described by Westergreen and Krasse. The adherent colonies of ms were observed by transillumination in a Spencer magnification lens (10 x). The total number of Streptococci colonies present in the Petri plate where obtained using the dilution coefficient and were called colony-forming units per mL of saliva (CFU/mL). As for *C. albicans*, they were obtained in agar Sabouraud, which were incubated in aerobic conditions for 48 h to 37 o (20).

- Biochemical study

The biochemical identification of *S. mutans*, or *S.sobrinus* was done by inoculating 2 colonies in the Todd Hewitt broth [3.1 g Brain Heart Infusion, 20g Peptone,2g Glucose, 2g Na Cl 0,4g Na2 HPO4, 2,5g Na2 CO3] during 18 hours. Bacteria were collected by centrifugation at 5,000 rpm. for 5min.The pellet was resuspended in 0,2 M Na2 HPO4 (7,4) buffer at N0 5 Mac Farland units (1.5x 109 CFU). This suspension was used to identify biotypes of smg through the following micro method: in a sterile plastic box was place a rectangle glass of 11.5 x 8 cm, in 48 pieces of 0.8cm in diameter each one, and the 30 μl suspension for carbohydrate was incubated in a cell culture chamber for 18 hours. The yeasts were identified as *C. albicans* based on formation of germinal tube that appears in human plasma when the cell incubate for 2 hours 37°C and carbohydrate assimilation test (20,21).

- Scanning electron microscopy

To reveal the adhesion of ms to yeast cells of *C. albicans*, the method described by Holmes *et al* was used. Cellular aggregations, previously centrifuged (6000 X5 min) were set Glutaraldehyde to 2.5% (vol/vol) in buffer of sodium cacodilato at 0.1 M at 4°C (pH 7.4) for 90 min. Cells were harvested by centrifugation for 1 min at 12000 x g and washed 4 times in 0.1 M sodium cacodylate. After this, cell were fixed with 1% osmium tetroxide at 20o C (1 h). Samples were dehydrated in different serial concentration of ethanol (30, 50,70, 95 an 100%) and dried with CO2 using a critical point apparatus (Polaron England) Samples were examined in a Zeiss DSM 940 (15 KV) (20).

- Serological study

It was performed using the double immunodiffusion technique described by Ouchterlony. The antiserum was prepared in female rabbits immunized with *S. mutans* (Ingbritt, serotype c) and *S. sobrinus* (OMZ 176 serotype d). The antigen was extracted by heat for 30 min at 60 º C (21-24). The strains were kindly provided by Professor Bratthall (RIP) of Sweden and Professor Loesche of the United States. For *C.albicans* was used as Reference strain ATCC 10231.

- Statistical analysis

To determine the differences between age groups in the count of *C. albicans*, ms and their serotypes, these groups where analyzed using one way ANOVA. In addition, the Scheffé test was used for comparison between groups and the non-paired T-Test to confront the control group with the Down syndrome Group. Previously the Shapiro-Wilks test was used to determine if there was a normal distribution of the sample. The analysis was performed with software Stata v 14.1 and it was considered that there were significant differences if *p* value < 0.05.

## Results

The sample analysis of Colony count/ml between CG and DS between *C. albicans* and ms proved to be significant for *C.albicans* (*P*<0,005) (Table 1).

**Figure 1:**
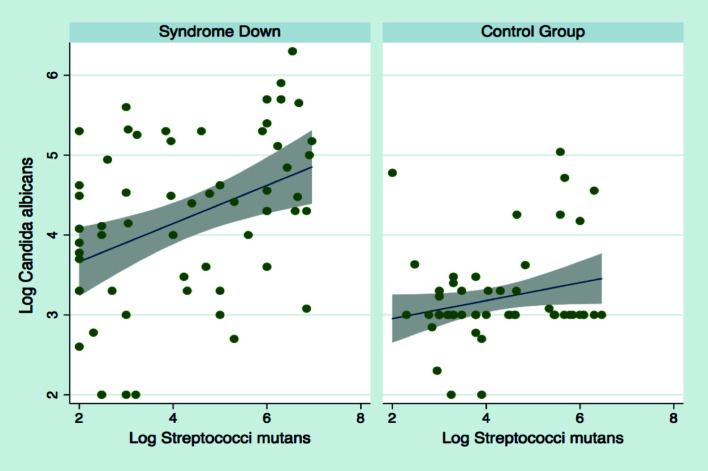
The patients with Down Syndrome have a highest number of log *C.albicans* and of the log of ms (colonies/ml) in saliva, this is evidenced with greater slope than in the healthy individuals of the CG.

When performing the linear regression analysis between patients and comparing the log of *C. albicans* versus log ms, and confronting these slopes of both groups CG vs DS, we observed a significant differences (*p*<0,005) (Fig. 1). Patients with Down Syndrome presented higher numbers (colonies/ml of saliva) of *C. albicans* and ms (colonies/ml saliva) than the CG in their respective means of cultivation of the same saliva sample.

Comparison of the average *C. albicans* and ms (log/ml) acording to age between control group and Down Syndrome finding significant different for ms GC and Down Syndrome groups age between 5 to 9 and 10 to 14 and 10 to 14 and 15 to 19 years (*P*<0,005) and (*P*<0,002) (Table 2), there were no significant differences in counting in for *C. albicans* in GC and SD.

**Table 1:**
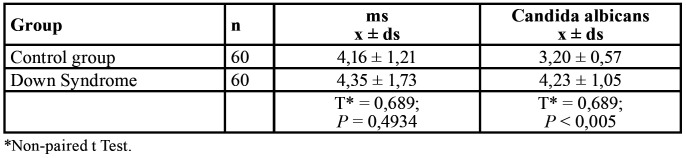
Comparison of the log count in saliva (colonies/ml) in Control group and Down Syndrome, between *mutans streptococci* and *Candida albicans*.

**Table 2:**
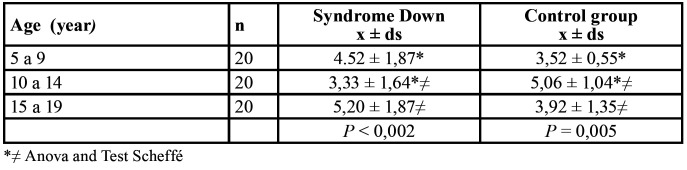
Comparison of saliva (colonies/ml) de ms (log) in Syndrome Down and control groups.

There were not significant age differences for C albicans in CG and SD according ages in groups 5 to 9 and 10 to 14 and between 10 a 14 and 15 to 19 (Table 3).

Analyzed the average count for *C. albicans* found in the two groups of patients studied and the serotypes of the ms. (Table 4). When performing t-Test comparing both patient groups for serotypes, it was significant for serotypes c, e and f (*p* < 0,005) and serotypes d and g (*p*< 0,02). No-typed was only observed in DS.

**Figure 2:**
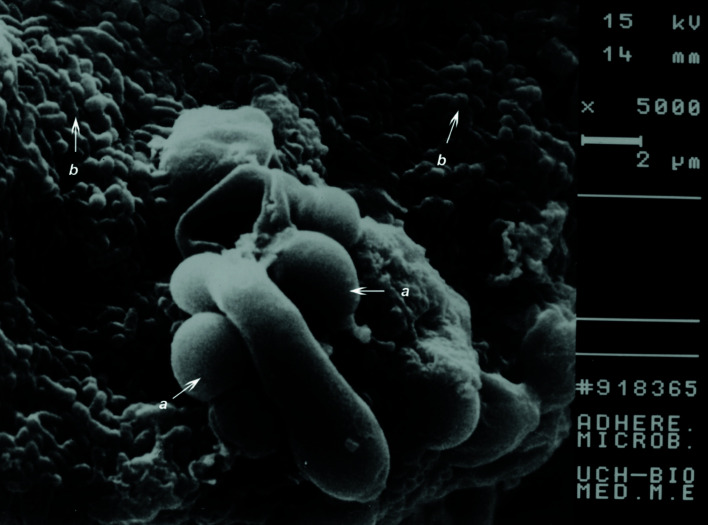
Scanning electron microscopy of a solid TYCSB culture for ms, where *Candida albicans* was developed. a) *Candida albicans*. b) ms.

It was observed through scanning electron microscopy (SEM), colonies in a sample of saliva of patients, using as culture medium TYCSB for the ms, standing out yuxtaposition of *C. albicans* in the culture medium (Fig. 2). When quantification was carried out in agar Saboureaud medium, an increase in the concentration was observed about between 104-105 Colonies/ml in saliva for C albicans, comparable to TYCSB that there was an increase in ms in patients with Down Syndrome (Fig. 2).

**Table 3:**
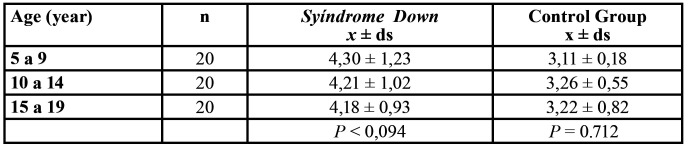
Comparison of *Candida albicans* saliva count (log) in Syndrome Down and Control Group.

**Table 4:**
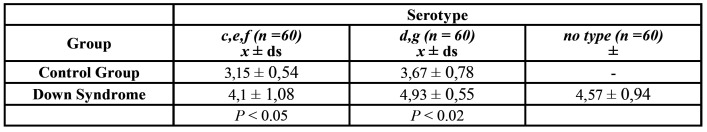
Average count of *Candida albicans* between patients in the control group (CG) and patients with Down Syndrome. Presents the serotypes for ms c, e, f, *S.sobrinus* d, g, and no type (only present in Down Syndrome).

## Discussion

Patients with Down Syndrome show a greater susceptibility to infections due to several factors that alter immune response as cellular and humoral (18,19). For this reason it is important to take into account the immune response in relation to oral infections, specifically interconnections between microorganisms involved in Caries and Periodontal disease in individuals who present these infectious pathologies, especially in the delivery of new properties that arise from these interrelationships or antagonism between the commensal microorganisms, which cannot be explained from an isolated cell and the possible impact on the patient's systemic level.

On the other hand,dental caries in children with mixed dentition is positively correlated with the frequency of oral candidal carriage (25).When interpreting the results obtained according to our hypothesis of the interrelation between C albicans and ms in saliva of patients in the DS and CG groups, it was evident that in the saliva cultures there were significant differences in the number of *C. albicans* in patients with DS (*P*< 0.005) (26) (Table 1). In other studies conducted in patients with Down syndrome and CG, they showed a high percentage of colonization for *C. albicans* of 69%, and 35% for the control group in children, to 7 months, 20 years 6 months (26). In Chile in patients with DS, similar results were obtained for *C. albicans* (20).

 The incidence of *C.albicans* isolated from the oral cavity, has been reported to be 45% in neonates, 45% a 60% of health children, 30%-45% healthy adults (27), interactions with bacterial present in saliva, as ms in the DS group. It could be through microbiological studies to study as *C.albicans* modulates its response from a physical, chemical and metabolic point of view in the presence of other microorganisms (9). Specifically to study the prevalence and possible pathogenicity mechanisms of it, against dental caries in children who are carriers of *C.albicans*, without leaving aside the set of environmental factors (25).

In both DS and CG groups the ms association was significant for DS, and minor in the control group (Fig. 1). Significant variations were found for ms in studied age groups (Table 2) but data show no significant diferences for *C. albicans* in both groups, (Table 3) but *C. albicans* contributes significantly to the capacity of the dental plaque community to cause disease (8).This would suggest that *C. albicans* could be a nursemaid cell for the ms group. The micro-colonies of *S.mutans* multiply quickly by doubling their size in the presence of *C. albicans* explaining the possible cause of early caries formation in children. The interaction between *C.albicans* and *S.mutans* is complex and have been studied *in vitro* model that establish explanations in relation to this duallity.The possible mechanism is that through the production of glucan by *S. mutans*, it binds to the cell wall of *C. albicans*, in the biofilm this supplies sites of adhesion resulting in a greater formation of biofilms of double species (28).

These biofilms of *C. albicans* and *S. mutans* give the possibility of increasing the production of exopolysaccharides (EPS), which allows retain the acid produced by *S.mutans*. In addition, this structure gives more support to biofilm by increasing the biomass, which only one species could give to house more *S.mutans* (29). On the other hand, an *in vitro* study contradicted that the colonization of *C. albicans* is concomitant with ms as a cariogenic factor, due to the increase in pH which prevents the loss of minerals (29). Thus *C.albicans* has often been associated with *S.mutans*, as they have created an acidogenic microenvironment, in an *in vitro* study of bacterial culture that incorporate hydroxyapatite discs in the presence of a biofilm of *C. albicans* and *S.mutans*, there was evidence of a decrease in the cariogenic potential in terms of acidification and demineralization, so it is proposed that *C.albicans* would have made a metabolic change like the consumption of oxygen and alkalization within the biofilm by consumption of lactic acid (29). Scientific evidence suggests that DS patients have lower caries prevalence than normal individuals, with risk to bias of reported evidence (14). A study in children with mixed dentition without DS showed that *C. albicans* has a higher prevalence in children with caries than in caries-free children (25). Another shows that *C. albicans* is associated with *S.mutans*, in the formation of early caries in children, which in older ages could be colonized only by *S.mutans*, present in the caries process (13).

With the identification of ms serotypes, it has been related that *S. sobrinus* is an aciduric species (Table 2) that is important in the caries of surfaces and rampant caries in children and adolescents with DS, which could be associated with *C. albicans* (12). In relation to serotypes, it was found that in *S. mutans* there is a different serotype corresponding to No- type (21), which could belong to the group k or other unknown serotype. This no-type serotype was evidenced in DS patients. The serotype No-type and c are in the same amount, while serotype d is in greater quantity (*p* < 0.02), being that serotype c the one that has the greatest presence in the human population (12). *C. albicans* would be significantly associated with the different serotypes of the ms present in humans. It demonstrated the relationship of a mutualism *in vivo* model between bacteria and fungus that has a clinically severe relevance ubiquitous in infectious diseases. On the other hand, Dentists and General Practitioner should be aware that oral disease can influence the systemic health (30).

Fig. 2 shows an *in vitro* interrelation between these microorganisms in a TYCSB medium in the groups studied . This medium is selective but not specific for the isolation of ms, and is used to correlate the degree of prevalence of dental caries in humans, being the *S. mutans* and *S. sobrinus* as initiators of enamel caries. The evidence shows that it could increase the number of ms colonies in this medium, and it makes possible the visualization of colonies of *C. albicans*.

In summary the present study shows, in saliva culture, the commensalism between *S. mutans* and *C. albicans* in the oral cavity among CG patients and DS. This commensalism would be an interaction between *C. albicans* and ms with different serotypes of the group.
